# Multiple Organ Dysfunction Interactions in Critically Ill Children

**DOI:** 10.3389/fped.2022.874282

**Published:** 2022-04-25

**Authors:** Colleen M. Badke, Anoop Mayampurath, L. Nelson Sanchez-Pinto

**Affiliations:** ^1^Division of Critical Care Medicine, Ann & Robert H. Lurie Children’s Hospital of Chicago, Chicago, IL, United States; ^2^Department of Pediatrics, Northwestern University Feinberg School of Medicine, Chicago, IL, United States; ^3^Stanley Manne Children’s Research Institute, Chicago, IL, United States; ^4^Department of Biostatistics and Medical Informatics, University of Wisconsin-Madison, Madison, WI, United States

**Keywords:** critical care, pediatrics, mortality, multiple organ dysfunction, data science

## Abstract

**Introduction:**

Multiple organ dysfunction (MOD) is a common pathway to morbidity and death in critically ill children. Defining organ dysfunction is challenging, as we lack a complete understanding of the complex pathobiology. Current pediatric organ dysfunction criteria assign the same diagnostic value—the same “weight”— to each organ system. While each organ dysfunction in isolation contributes to the outcome, there are likely complex interactions between multiple failing organs that are not simply additive.

**Objective:**

Determine whether certain combinations of organ system dysfunctions have a significant interaction associated with higher risk of morbidity or mortality in critically ill children.

**Methods:**

We conducted a retrospective observational cohort study of critically ill children at two large academic medical centers from 2010 and 2018. Patients were included in the study if they had at least two organ dysfunctions by day 3 of PICU admission based on the Pediatric Organ Dysfunction Information Update Mandate (PODIUM) criteria. Mortality was described as absolute number of deaths and mortality rate. Combinations of two pediatric organ dysfunctions were analyzed with interaction terms as independent variables and mortality or persistent MOD as the dependent variable in logistic regression models.

**Results:**

Overall, 7,897 patients met inclusion criteria and 446 patients (5.6%) died. The organ dysfunction interactions that were significantly associated with the highest absolute number of deaths were cardiovascular + endocrinologic, cardiovascular + neurologic, and cardiovascular + respiratory. Additionally, the interactions associated with the highest mortality rates were liver + cardiovascular, respiratory + hematologic, and respiratory + renal. Among patients with persistent MOD, the most common organ dysfunctions with significant interaction terms were neurologic + respiratory, hematologic + immunologic, and endocrinologic + respiratory. Further analysis using classification and regression trees (CART) demonstrated that the absence of respiratory and liver dysfunction was associated with the lowest likelihood of mortality.

**Implications and Future Directions:**

Certain combinations of organ dysfunctions are associated with a higher risk of persistent MOD or death. Notably, the three most common organ dysfunction interactions were associated with 75% of the mortality in our cohort. Critically ill children with MOD presenting with these combinations of organ dysfunctions warrant further study.

## Introduction

Multiple organ dysfunction (MOD) is a final common pathway for death and long-term morbidity in critically ill children for many etiologies and pathophysiologic processes (1). Approximately 20% of children have two or more organ dysfunctions at the time of pediatric intensive care unit (PICU) admission (2), while an additional 23% of patients develop new MOD during their PICU course (3). Given that PICU mortality rates are low, various definitions of organ dysfunction are often used as a surrogate for morbidity in the PICU (4). Additionally, survivors of MOD frequently suffer long-term functional impairment and disability (4–6). However, defining single and MODs is challenging, as we lack a complete understanding of the complex pathobiology underlying most cases of MOD (7).

Various diagnostic criteria for pediatric organ dysfunction have been developed, validated, and applied clinically, including those by Wilkinson, Proulx, and the International Pediatric Sepsis Consensus Conference (IPSCC) (8–10). Recently, the Pediatric Organ Dysfunction Information Update Mandate (PODIUM) investigators developed novel consensus criteria for single and MOD based on systematic literature reviews (11). These criteria were validated using electronic health record (EHR) data and they showed improved performance in discriminating mortality compared to the widely used IPSCC criteria for organ dysfunction (12). However, in the PODIUM criteria and in other MOD classification systems, the same “weight” is given to each organ system. While each organ dysfunction in isolation contributes to poor outcomes, there are likely complex pathophysiological interactions between failing organs with effects that are not simply additive. We do not know which combinations of organ dysfunction have the strongest association with poor outcomes. Identifying MOD patients with the most lethal combinations of organ dysfunctions may have important implications for focusing research efforts and personalizing clinical care. Therefore, the objective of this study was to use a data-driven approach to better describe the combinations of organ dysfunctions that are seen critically ill children with MOD, to evaluate the impact of interactions between organ dysfunctions, and to evaluate whether those interactions are significantly associated with persistent MOD or mortality in this population.

## Materials and Methods

### Study Design and Population

We conducted a retrospective observational cohort study of patients aged 0–17 years of age admitted to the PICU between 2010 and 2018 (Ann & Robert H. Lurie Children’s Hospital of Chicago) or between 2010 and 2016 (University of Chicago Comer Children’s Hospital) who had at least two organ dysfunctions by day 3 of PICU admission. Patients were excluded if they had congenital heart disease or if their primary reason for admission was cardiac surgery. Data were extracted from the two institutional EHR databases using structured queries and underwent quality checks for conformity, completeness, and plausibility (13). Only data from the first PICU encounter in each hospitalization was included. Each hospitalization was treated independently. The Institutional Review Boards at Ann & Robert H. Lurie Children’s Hospital of Chicago and The University of Chicago approved this study with a waiver of informed consent.

### Definitions of Organ Dysfunction

We considered nine different organ dysfunctions in all patients per the PODIUM consensus criteria (cardiovascular, coagulation, respiratory, neurologic, renal, hematologic, immunologic, liver, and endocrinologic). The methods used to calculate these organ dysfunctions from EHR data have been previously published (11). Missing values were assumed to be within the normal range, and therefore negative for organ dysfunction.

### Outcomes

We assessed two primary outcomes: development of persistent MOD and death. Death was defined as in-hospital mortality and classified as absolute number of deaths and mortality rate. MOD was defined as the presence of ≥2 concurrent organ dysfunctions regardless of cause, and persistent MOD was defined as the presence of MOD on day 7 after PICU admission (14–16).

### Statistical Analysis and Classification and Regression Trees

Data were analyzed using R version 4.0 (R Foundation for Statistical Computing, Vienna, Austria) (17). Interaction for combinations of 2 or 3 organ dysfunctions was analyzed using interaction terms in logistic regression models, with a *p*-value < 0.005 considered significant (18). We did not examine interactions between coagulation and hematologic dysfunction or coagulation and liver dysfunction given the overlap in their PODIUM diagnostic criteria.

Organ dysfunctions were then incorporated as predictors in a Classification and Regression Tree (CART) model for the outcomes of mortality or persistent MOD to study the non-linear relationship between MOD combinations. The model was derived using 80% of patients in the cohort and validated in the remaining 20%. We used 10-fold cross-validation of the derivation set to prune and fit the CART model. Weighting of cases and costs for misclassification were not used. The performance of this model was assessed using the area under the receiver operating characteristic curve (AUROC).

## Results

Overall, 7,897 patients among the two PICU populations met the inclusion criteria. The median age on admission was 6.5 years (interquartile range [IQR] 1.6–13.0). Across both centers, 446 patients (5.6%) died, while 1,002 patients (12.7%) were alive with persistent organ dysfunction by day 7. We examined the incidence of nine different organ dysfunctions in all patients and in patients who died (Table 1). The most common organ dysfunctions among all patients were endocrinologic (61%), cardiovascular (51%), immunologic (49%), and neurologic (48%). However, non-survivors had a greater proportion of liver (28%), coagulation (15%), respiratory (11%), and hematologic (10%) dysfunction (Table 1). Organ dysfunction severity by 28 days is describe in Supplementary Table 1.

**TABLE 1:** Demographics of patients with organ dysfunction by day 3 of PICU admission.

	All patients (*n* = 7897)	Survivors (*n* = 7431)	Non-survivors (*n* = 466)	*p*-value**
Age, median [IQR*] (years)	6.5 (1.6–13.0)	6.5 (1.6–13.0)	4.7 (1.0–11.9)	0.002
Sex, male (*n*, %)	4274 (54.1%)	4014 (54.0%)	260 (55.8%)	0.48
Race				
Black/African American	2586 (32.7%)	2440 (32.8%)	146 (31.3%)	0.04
White	2536 (32.1%)	2392 (32.35)	144 (30.9%)	
Hispanic/Latino	2021 (25.6%)	1907 (25.7%)	114 (24.5%)	
Other/Unknown	754 (9.5%)	692 (9.3%)	62 (13.3%)	
LOS, median [IQR] (days)	7.7 (4.1–15.1)	7.8 (4.3–15.1)	4.3 (1.5–19.0)	<0.001
Mechanical ventilation days, median [IQR]	1.0 (0–5.0)	1.0 (0.0–5.0)	4.0 (1.0–13.0)	<0.001
PRISM III score, median (IQR)	6.0 (2.0–11.0)	6.0 (2.0–10.0)	19.0 (9.0–28.0)	<0.001
Organ dysfunction incidence by day 3 (*n*, %)				
Endocrinologic	4790 (60.7%)	4414 (59.4%)	376 (80.7%)	<0.001
Cardiovascular	4015 (50.8%)	3634 (48.9%)	381 (81.8%)	<0.001
Immunologic	3878 (49.1%)	3617 (48.7%)	261 (56.0%)	0.002
Neurologic	3800 (48.1%)	3440 (46.3%)	360 (77.3%)	<0.001
Respiratory	3292 (41.7%)	2933 (39.5%)	359 (77.0%)	<0.001
Hematologic	2799 (35.4%)	2519 (33.9%)	280 (60.1%)	<0.001
Renal	2505 (31.7%)	2273 (30.6%)	232 (49.8%)	<0.001
Coagulation	808 (10.2%)	689 (9.3%)	119 (25.5%)	<0.001
Liver	562 (7.1%)	403 (5.4%)	159 (34.1%)	<0.001

*IQR, interquartile range.

**Chi-squared performed for categorical variables.

*Kruskal–Wallis performed for continuous variables.*

*p-value > 0.05 considered significant.*

### Interaction Analysis

#### Mortality

**FIGURE 1:**
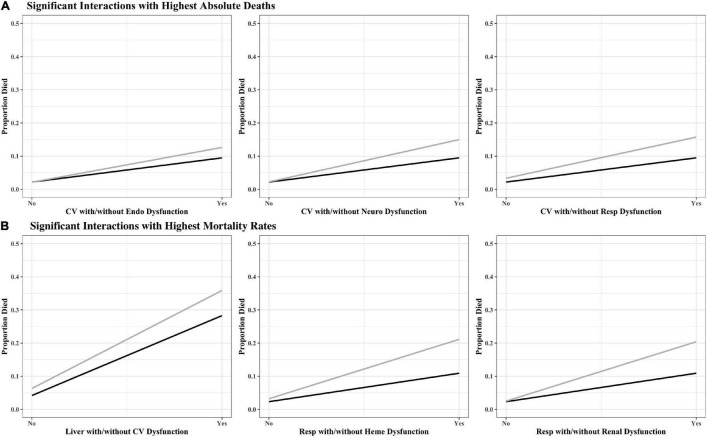
Interaction plots for high-risk organ dysfunction pairs associated with **(A)** the highest number of absolute deaths and **(B)** highest mortality rates. The black line represents single organ dysfunction, while the gray line represents the interaction with the second organ dysfunction. Coag, coagulation; CV, cardiovascular; Endo, endocrinologic; Immuno, immunologic; Heme, hematologic; Neuro, neurologic; Resp, respiratory.

**FIGURE 2:**
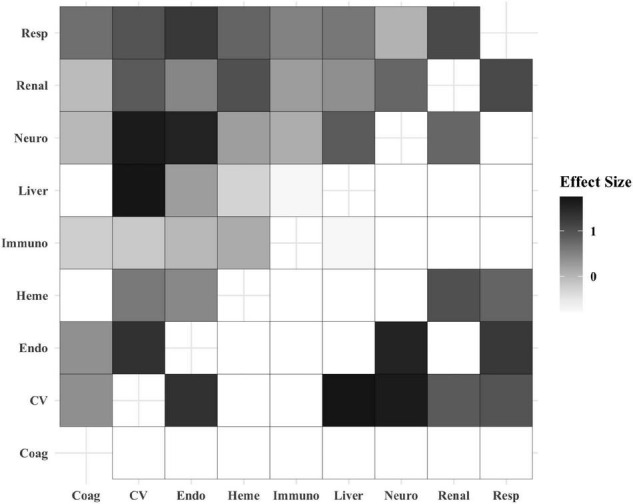
Heatmap displaying the effect size of organ dysfunction interactions and association with mortality. The boxes in the top-left half of the heatmap include the effect size for all of the organ dysfunction interactions based on their coefficients. The boxes in the bottom-right half of the heatmap display only the effect size for the statistically significant organ dysfunction interactions. The white boxes in this half of the heatmap were non-significant and therefore not shaded. Increased box shading indicates a stronger effect size. Coag, coagulation; CV, cardiovascular; Endo, endocrinologic; Immuno, immunologic; Heme, hematologic; Neuro, neurologic; Resp, respiratory.

The organ dysfunction interactions that were significantly associated with the highest absolute number of deaths were cardiovascular + endocrinologic (329 deaths; interaction *p* < 0.001), cardiovascular + neurologic (323 deaths; *p* < 0.001) and cardiovascular + respiratory (316 deaths; *p* < 0.001), while the interactions associated with the highest mortality rates were liver + cardiovascular (35.9% mortality; *p* = 0.001), respiratory + hematologic (21.1% mortality; *p* < 0.001), and respiratory + renal (17.5% mortality; *p* < 0.001) (Table 2 and Figure 1). The effect sizes for organ dysfunction interactions associated with mortality are displayed in Figure 2. The result for all combinations of two organ dysfunction interactions are presented in Supplementary Table 2 and Supplementary Figure 1. The median length of stay for survivors with these organ dysfunction interactions was: 10.0 days (IQR 4.9–20.4) for cardiovascular + endocrinologic; 13.3 days (IQR 7.0–24.9) for cardiovascular + neurologic; 14.1 days (IQR 7.8–26.4) for cardiovascular + respiratory; 22.9 days (IQR 12.9–41.4) for liver + cardiovascular; 18.0 days (IQR 10.3–31.5) for respiratory + hematologic; and 16.3 days (IQR 9.8–32.1) for respiratory + renal. When examining interactions for patient with three organ dysfunctions, two interaction terms were significantly associated with mortality: endocrinologic + immunologic + neurologic (164 deaths [15.6% mortality]; *p* = 0.003) and endocrinologic + immunologic + respiratory (171 deaths [17.2%]; *p* = 0.002) (Supplementary Table 3).

**TABLE 2:** Top three organ dysfunction interactions associated with increased mortality, by **(A)** absolute number of deaths and **(B)** mortality rate.

	A. Highest absolute number of deaths, *N* (%)			B. Highest mortality rate, *N* (%)		
	2-Way Interactions	Total *N* (%)	Died *N* (%)	2-Way Interactions	Total *N* (%)	Died *N* (%)
1.	CV + Endo	2603 (33.0%)	329 (12.6%)	Liver + CV	432 (5.5%)	155 (35.9%)
2.	CV + Neuro	2154 (27.3%)	323 (15.0%)	Resp + Heme	1060 (13.4%)	224 (21.1%)
3.	CV + Resp	2007 (25.4%)	316 (15.7%)	Resp + Renal	964 (12.2%)	169 (17.5%)

*N = 7897.*

#### Persistent Multiple Organ Dysfunction

Among patients who had persistent MOD, the most common organ dysfunctions with significant interaction terms were neurologic + respiratory (557 patients [55.6% of persistent MOD]; interaction *p* < 0.001), hematologic + immunologic (492 patients [49.1%]; *p* = 0.007), and endocrinologic + respiratory (484 patients [48.3%]; *p* < 0.001) (Supplementary Table 2). The median length of stay for patients with these organ dysfunction interactions was: 14.0 days (IQR 7.8–25.5) for neurologic + respiratory; 9.7 days (IQR 5.4–18.4) for hematologic + immunologic; and 14.0 days (IQR 7.8–25.9) for endocrinologic + respiratory. Additionally, we examined interactions for patients with three organ dysfunctions. The most frequent organ dysfunctions that had significant interaction terms were endocrinologic + neurologic + respiratory (422 patients [42.1%]; *p* = 0.005), cardiovascular + hematologic + immunologic (279 patients [27.8%]; *p* < 0.001), and endocrinologic + renal + respiratory (215 patients [21.5%]; *p* = 0.009).

### Classification and Regression Trees Model

**FIGURE 3:**
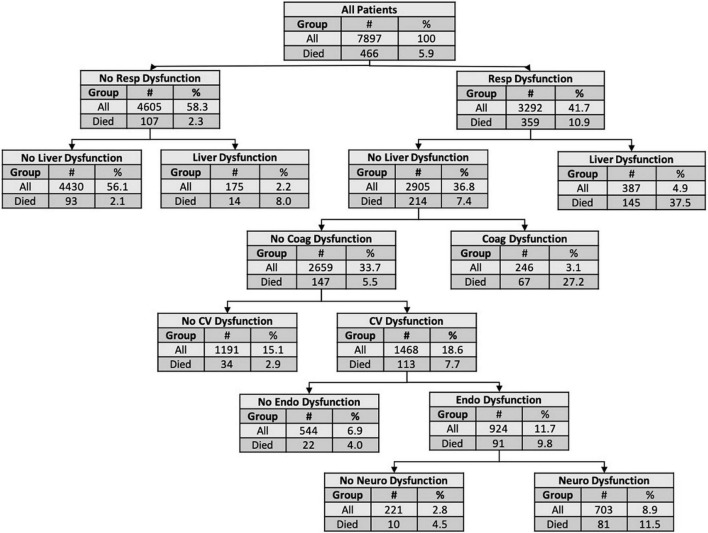
Classification and regression tree for mortality. The nodes and leaves were based on 10-fold cross validation in the derivation set but the numbers presented are for the entire cohort. The top node of the decision tree (the root node) shows the total number of subjects in the cohort and the number and proportion of non-survivors. Each subsequent node shows the criterion for subsequent decisions, along with the number and proportion of patients with or without the organ dysfunction and the proportion who died. Terminal nodes show the risk for an individual with the preceding organ dysfunctions. AUROC: 0.75 (95% CI 0.69–0.82). Coag, coagulation; CV, cardiovascular; Endo, endocrinologic; Neuro, neurologic; Resp, respiratory.

The CART model for mortality is shown in Figure 3. This model discriminated death with an AUROC of 0.75 (95% confidence interval [CI] 0.69–0.82) in the validation set. The organ dysfunctions closest to the root node and with the strongest impact in the CART model for mortality were respiratory and liver. The CART model for persistent MOD performed with an AUROC of 0.74 (95% CI 0.70–0.78) in the validation set (Supplementary Figure 2). The organ dysfunctions with the strongest impact in the CART model for persistent MOD were hematologic and respiratory.

## Discussion

Critically ill children with single organ dysfunction are at risk of developing MOD, persistent MOD, and death (15). Additionally, certain phenotypes of MOD are associated with increased risk of morbidity and mortality (14). In this study, we demonstrate that certain organ dysfunctions significantly interact with each other, increasing the risk for persistent MOD and death. The three most common significant organ dysfunction interactions associated with mortality (cardiovascular + liver, respiratory + hematologic, and respiratory + renal dysfunctions) were associated with 75% of the deaths in our cohort. Additionally, the three most common significant organ dysfunction interactions associated with persistent MOD (neurologic + respiratory, hematologic + immunologic, and endocrinologic + respiratory) were associated with 36.3% of the cases of persistent MOD.

Many of the interactions detailed in our results have been individually described in the literature, with some studies evaluating targeted therapies for specific phenotypes characterized by organ dysfunction combinations. For example, liver with cardiovascular dysfunction has been described in the sepsis-associated macrophage activation syndrome (MAS) phenotype, characterized by shock, hepatobiliary dysfunction, and disseminated intravascular coagulation (DIC) (19). In these high-risk patients with MAS, targeted anti-cytokine therapies have been shown to reduce mortality (20). Another high-risk organ dysfunction interaction in our cohort, respiratory and renal dysfunction, has been described in critically ill children, particularly in the context of fluid overload. Existing literature has linked fluid overload with respiratory failure (particularly in the form of increased ventilator days) and mortality (21, 22).

Classification and regression trees models have previously been used to reveal non-linear interactions between variables in disease states like pediatric sepsis, acute respiratory distress syndrome, and acute kidney injury (23–25). Our CART analysis illustrates which organ dysfunctions tend to drive the association with the outcome. To our knowledge, this is the first CART analysis examining interactions in pediatric MOD. For non-survivors in our cohort, respiratory and liver dysfunction were shown drive the association with mortality, as these were the first nodes in the classification tree. For example, patients with MOD that have no respiratory or liver dysfunction in the first 3 days of PICU admission have the lowest risk for death at 2% mortality, compared to patients with liver but no respiratory dysfunction (8%), respiratory but no liver dysfunction (7%) or with both dysfunctions (38%). For persistent MOD, the association was driven by hematologic and respiratory dysfunction. Critically ill children without hematologic or respiratory dysfunction in the first 3 days of admission have the lowest risk for developing persistent MOD (2%) compared to when both dysfunctions are present (34%).

Our study has several strengths and limitations. Patient data was extracted from EHR databases corresponding to two large, urban, academic PICUs. The size and heterogeneity of the patient population is an advantage from a generalizability standpoint. However, our cohort may not be representative of all PICU populations, particularly in smaller, less urban, or non-academic settings. Expanding to other centers would provide us with increased power to detect other statistically significant interactions in organ dysfunction and death. Additionally, this retrospective study only describes the association of organ dysfunction interactions that occur within 3 days of PICU admission; we were unable to link this with admission diagnoses or assess longitudinal change in organ dysfunction. For example, if a patient is admitted with cardiovascular dysfunction and later develops liver dysfunction, is that patient’s trajectory different from someone who first develops liver dysfunction, followed by cardiovascular dysfunction? Similarly, the PODIUM criteria do not differentiate between acute or chronic organ dysfunction. It is plausible that patients with existing, chronic organ dysfunction who then develop acute-on-chronic dysfunction may have a higher risk for a poor outcome compared to patients with only acute organ dysfunctions. As our diagnostic tools improve, criteria like PODIUM will be better equipped to diagnose organ dysfunction in a timely and specific way. Third, our analysis did not control for factors such as age, comorbidities, or genetic factors, although age is accounted for in the PODIUM consensus criteria. It is plausible that these factors could be influential in organ dysfunction interactions, especially related to progressive morbidity or mortality (26).

In conclusion, organ dysfunction interactions occur frequently in critically ill children and specific interactions are significantly associated with persistent MOD and mortality. Patients presenting with these high-risk organ dysfunction combinations early in their PICU course warrant additional study, as earlier identification of these high-risk patients may impact treatment decisions and clinical outcomes. The high absolute number of deaths associated with certain organ dysfunction combinations warrant further focused research efforts, as targeted interventions on these patients may be able to have a higher clinical impact. Additionally, understanding which organ dysfunction interactions are associated with the highest risk for death may enhance situational awareness and prioritize resource utilization in the intensive care unit, in addition to supporting prognostication of outcomes at the patient level.

## Data Availability Statement

The raw data supporting the conclusions of this article will be made available by the authors, without undue reservation.

## Ethics Statement

The studies involving human participants were reviewed and approved by Ann & Robert H. Lurie Children’s Hospital of Chicago Institutional Review Board. Written informed consent from the participants’ legal guardian/next of kin was not required to participate in this study in accordance with the national legislation and the institutional requirements.

## Author Contributions

CB and LS-P designed the study and performed the data analysis. All authors made substantial contributions to drafting and final approval of the manuscript, and agree to be accountable for the content of the work.

## Conflict of Interest

The authors declare that the research was conducted in the absence of any commercial or financial relationships that could be construed as a potential conflict of interest.

## Publisher’s Note

All claims expressed in this article are solely those of the authors and do not necessarily represent those of their affiliated organizations, or those of the publisher, the editors and the reviewers. Any product that may be evaluated in this article, or claim that may be made by its manufacturer, is not guaranteed or endorsed by the publisher.

## Abbreviations

CART: classification and regression tree
EHR: electronic health record
IPSCC: International Pediatric Sepsis Consensus Conference
MOD: multiple organ dysfunction
PICU: pediatric intensive care unit
PODIUM: pediatric organ dysfunction information update mandate
pSOFA: pediatric sequential organ failure assessment.
