# Supplementary material for: Multiple Organ Dysfunction Interactions in Critically Ill Children
Source: Front Pediatr. 2022 Apr 25;10:874282. doi: 10.3389/fped.2022.874282 (PMC9081807; doi:10.3389/fped.2022.874282)
Supplement: Supplementary file 1 [file Data_Sheet_1.pdf]

Supplementary Material

Supplementary Table 1. Presence of organ dysfunction and severity of dysfunction* for survivors and non-survivors by 28 days.

Organ Dysfunction Incidence by Day 28 (n, %)	All Patients (n=7897)	Survivors (n=7431)	Non-survivors (n=466)
**Endocrinologic**	5143 (65.1%)	4730 (62.4%)	413 (88.6%)
**Cardiovascular**	4384 (55.5%)	3957 (53.2%)	427 (91.6%)
**Severe Cardiovascular**	881 (11.2%)	617 (8.3%)	264 (56.7%)
**Immunologic**	4184 (53.0%)	3901 (52.5%)	283 (60.7%)
**Neurologic**	4156 (52.6%)	3742 (50.4%)	414 (88.8%)
**Respiratory**	3555 (45.0%)	3158 (42.5%)	397 (85.2%)
**Severe Respiratory**	1009 (12.8%)	750 (10.1%)	259 (55.6%)
**Hematologic**	3204 (40.6%)	2879 (38.7%)	325 (69.7%)
**Severe Hematologic**	351 (4.4%)	304 (4.1%)	47 (10.1%)
**Renal**	2898 (36.7%)	2620 (35.3%)	278 (59.7%)
**Coagulation**	994 (12.6%)	835 (11.2%)	159 (34.1%)
**Liver**	623 (7.9%)	437 (5.9%)	186 (39.9%)

*Severity grading based on PODIUM criteria is only available for cardiovascular, respiratory, and hematologic dysfunction

Supplementary Table 2. Association between two organ dysfunction interactions and mortality or persistent MOD.

Combinations of two Organ Dysfunctions	Total (n, %)	Died (n=466)	p-value	Persistent MOD (n=1002)	p-value
**Coag+CV**	518 (6.6%)	107 (20.7%)	0.26	166 (16.6%)	0.13
**Coag+Endo**	597 (7.6%)	107 (17.9%)	0.27	200 (20.0%)	0.15
**Coag+Immuno**	553 (7.0%)	81 (14.6%)	0.34	191 (19.1%)	0.86
**Coag+Neuro**	370 (4.7%)	88 (23.8%)	0.99	145 (14.5%)	0.89
**Coag+Renal**	354 (4.5%)	70 (19.8%)	0.91	137 (13.7%)	0.06
**Coag+Resp**	379 (4.8%)	102 (26.9%)	0.02	160 (16.0%)	0.89
**CV+Endo**	2603 (33.0%)	329 (12.6%)	<0.001*	476 (47.5%)	<0.001*
**CV+Heme**	1419 (18.0%)	237 (16.7%)	0.02	397 (39.6%)	0.86
**CV+Immuno**	1728 (21.9%)	202 (11.7%)	0.51	382 (38.1%)	0.54
**CV+Liver**	432 (5.5%)	155 (35.9%)	0.001*	140 (14.0%)	0.02
**CV+Neuro**	2154 (27.3%)	323 (15.0%)	<0.001*	472 (47.1%)	<0.001*
**CV+Renal**	1240 (15.7%)	202 (16.3%)	<0.001*	268 (26.7%)	0.14
**CV+Resp**	2007 (25.4%)	316 (15.7%)	<0.001*	480 (47.9%)	<0.001*
**Endo+Heme**	1683 (21.3%)	232 (13.8%)	0.06	466 (46.5%)	0.003*
**Endo+Immuno**	2399 (30.4%)	212 (8.8%)	0.98	469 (46.8%)	0.12
**Endo+Liver**	491 (6.2%)	149 (30.3%)	0.50	164 (16.4%)	0.79
**Endo+Neuro**	2258 (28.6%)	310 (13.7%)	<0.001*	480 (47.9%)	<0.001*
**Endo+Renal**	1642 (20.8%)	200 (12.2%)	0.05	321 (32.0%)	0.10
**Endo+Resp**	2001 (25.3%)	307 (15.3%)	<0.001*	484 (48.3%)	<0.001*
**Heme+Immuno**	1833 (23.2%)	188 (10.3%)	0.56	492 (49.1%)	0.007
**Heme+Liver**	433 (5.5%)	131 (30.3%)	0.31	161 (16.1%)	0.28
**Heme+Neuro**	1164 (14.7%)	211 (18.1%)	0.30	349 (34.8%)	<0.001*
**Heme+Renal**	964 (12.2%)	169 (17.5%)	<0.001*	295 (29.4%)	0.03
**Heme+Resp**	1060 (13.4%)	224 (21.1%)	<0.001*	360 (35.9%)	<0.001*
**Immuno+Liver**	329 (4.2%)	83 (25.2%)	<0.001*	126 (12.6%)	0.58
**Immuno+Neuro**	1522 (19.3%)	189 (12.4%)	0.68	355 (35.4%)	<0.001*
**Immuno+Renal**	1165 (14.8%)	132 (11.3%)	0.18	303 (30.2%)	<0.001*
**Immuno+Resp**	1376 (17.4%)	200 (14.5%)	0.02	359 (35.8%)	<0.001*
**Liver+Neuro**	439 (5.6%)	150 (34.2%)	0.02	147 (14.7%)	0.03
**Liver+Renal**	323 (4.1%)	113 (35.0%)	0.09	118 (11.8%)	0.92

**Liver+Resp**	387 (4.9%)	145 (37.5%)	0.06	148 (14.8%)	0.64
**Neuro+Renal**	1119 (14.2%)	189 (16.9%)	<0.001*	273 (27.2%)	0.03
**Neuro+Resp**	2296 (29.1%)	303 (13.2%)	0.81	557 (55.6%)	<0.001*
**Renal+Resp**	946 (12.0%)	193 (20.4%)	<0.001*	267 (26.6%)	0.09

*p<0.005 considered statistically significant.

Coag: coagulation
CV: cardiovascular
Endo: endocrinologic
Immuno: immunologic
Heme: hematologic
Neuro: neurologic
Resp: respiratory

Supplementary Table 3. Association between three organ dysfunction interactions and mortality or persistent MOD.

Combined Organ Dysfunctions	All Patients (n=7897)	Died (n=466)	p-value	Persistent MOD (n=1002)	p-value
**Coag+CV+Endo**	420 (5.3%)	100 (23.8%)	0.72	146 (14.6%)	0.67
**Coag+CV+Immuno**	356 (4.5%)	70 (19.7%)	0.11	127 (12.7%)	0.30
**Coag+CV+Neuro**	294 (3.7%)	86 (29.3%)	0.72	119 (11.9%)	0.17
**Coag+CV+Renal**	250 (3.2%)	64 (25.6%)	0.14	98 (9.8%)	0.44
**Coag+CV+Resp**	312 (4.0%)	95 (30.4%)	0.41	132 (13.2%)	0.34
**Coag+Endo+Immuno**	422 (5.3%)	73 (17.3%)	0.68	158 (15.8%)	0.70
**Coag+Endo+Neuro**	322 (4.1%)	84 (26.1%)	0.27	128 (12.8%)	0.003*
**Coag+Endo+Renal**	288 (3.6%)	65 (22.6%)	0.64	115 (11.5%)	0.12
**Coag+Endo+Resp**	327 (4.1%)	96 (29.4%)	0.93	141 (14.1%)	0.04
**Coag+Immuno+Neuro**	247 (3.1%)	57 (23.1%)	0.23	110 (11.0%)	0.04
**Coag+Immuno+Renal**	249 (3.2%)	49 (19.7%)	0.74	108 (10.8%)	0.05
**Coag+Immuno+Resp**	264 (3.3%)	69 (26.1%)	0.22	118 (11.8%)	0.89
**Coag+Neuro+Renal**	198 (2.5%)	54 (27.3%)	0.02	94 (9.4%)	0.53
**Coag+Neuro+Resp**	292 (3.7%)	85 (29.1%)	0.40	130 (13.0%)	0.004*
**Coag+Renal+Resp**	211 (2.7%)	63 (29.9%)	0.05	97 (9.7%)	0.14
**CV+Endo+Heme**	1026 (13.0%)	213 (20.8%)	0.18	322 (32.1%)	0.73
**CV+Endo+Immuno**	1217 (15.4%)	179 (14.7%)	0.71	311 (31.0%)	0.27
**CV+Endo+Liver**	387 (4.9%)	146 (37.7%)	0.93	130 (13.0%)	0.54
**CV+Endo+Neuro**	1488 (18.8%)	289 (19.4%)	0.61	374 (37.3%)	0.03
**CV+Endo+Renal**	912 (11.5%)	183 (20.1%)	0.59	221 (22.1%)	0.49
**CV+Endo+Resp**	1414 (17.9%)	285 (20.2%)	0.14	377 (37.6%)	<0.001*
**CV+Heme+Immuno**	890 (11.3%)	152 (17.1%)	0.24	279 (27.8%)	<0.001*
**CV+Heme+Liver**	336 (4.3%)	127 (37.8%)	0.96	122 (12.2%)	0.52
**CV+Heme+Neuro**	816 (10.3%)	201 (24.6%)	0.35	275 (27.4%)	0.29
**CV+Heme+Renal**	573 (7.3%)	156 (27.2%)	0.04	183 (18.3%)	<0.001*
**CV+Heme+Resp**	795 (10.1%)	206 (25.9%)	0.79	288 (28.7%)	0.63
**CV+Immuno+Liver**	249 (3.2%)	80 (32.1%)	0.43	96 (9.6%)	0.63
**CV+Immuno+Neuro**	897 (11.4%)	167 (18.6%)	0.28	266 (26.5%)	0.86
**CV+Immuno+Renal**	562 (7.1%)	113 (20.1%)	0.78	180 (18.0%)	0.01
**CV+Immuno+Resp**	879 (11.1%)	172 (19.6%)	0.60	274 (27.3%)	0.77
**CV+Liver+Neuro**	369 (4.7%)	148 (40.1%)	0.61	121 (12.1%)	0.01
**CV+Liver+Renal**	273 (3.5%)	112 (41.0%)	0.51	96 (9.6%)	0.30

**CV+Liver+Resp**	341 (4.3%)	142 (41.6%)	0.41	122 (12.2%)	0.001*
**CV+Neuro+Renal**	728 (9.2%)	176 (24.2%)	0.37	218 (21.8%)	0.24
**CV+Neuro+Resp**	1469 (18.6%)	283 (19.3%)	0.02	428 (42.7%)	0.77
**CV+Renal+Resp**	679 (8.6%)	178 (26.2%)	0.76	215 (21.5%)	1.0
**Endo+Heme+Immuno**	1073 (13.6%)	152 (14.2%)	0.39	352 (35.1%)	0.79
**Endo+Heme+Liver**	381 (4.8%)	124 (32.5%)	0.48	144 (14.4%)	0.65
**Endo+Heme+Neuro**	864 (10.9%)	188 (21.8%)	0.01	289 (28.8%)	0.43
**Endo+Heme+Renal**	693 (8.8%)	153 (22.1%)	0.05	234 (23.4%)	0.69
**Endo+Heme+Resp**	806 (10.2%)	198 (24.6%)	0.21	296 (29.5%)	0.18
**Endo+Immuno+Liver**	295 (3.7%)	79 (26.8%)	0.90	116 (11.6%)	0.50
**Endo+Immuno+Neuro**	1048 (13.3%)	164 (15.6%)	0.003*	290 (28.9%)	0.93
**Endo+Immuno+Renal**	813 (10.3%)	118 (14.5%)	0.23	233 (23.3%)	0.32
**Endo+Immuno+Resp**	995 (12.6%)	171 (17.2%)	0.002*	289 (28.8%)	0.02
**Endo+Liver+Neuro**	395 (5.0%)	141 (35.7%)	0.23	132 (13.2%)	0.009
**Endo+Liver+Renal**	294 (3.7%)	106 (36.1%)	0.23	109 (10.9%)	0.91
**Endo+Liver+Resp**	355 (4.5%)	138 (38.9%)	0.77	133 (13.3%)	0.003*
**Endo+Neuro+Renal**	755 (9.6%)	170 (22.5%)	0.87	216 (21.6%)	0.09
**Endo+Neuro+Resp**	1474 (18.7%)	274 (18.6%)	0.03	422 (42.1%)	0.005
**Endo+Renal+Resp**	695 (8.8%)	174 (25.0%)	0.97	215 (21.5%)	0.009
**Heme+Immuno+Liver**	281 (3.6%)	75 (26.7%)	0.42	115 (11.5%)	0.29
**Heme+Immuno+Neuro**	659 (8.3%)	135 (20.5%)	0.89	234 (23.4%)	0.88
**Heme+Immuno+Renal**	605 (7.7%)	112 (18.5%)	0.38	230 (23.0%)	0.17
**Heme+Immuno+Resp**	619 (7.8%)	146 (23.6%)	0.30	239 (23.9%)	0.13
**Heme+Liver+Neuro**	347 (4.4%)	124 (35.7%)	0.91	127 (12.7%)	0.73
**Heme+Liver+Renal**	268 (3.4%)	101 (37.7%)	0.52	102 (10.2%)	0.003*
**Heme+Liver+Resp**	316 (4.0%)	122 (38.6%)	0.90	128 (12.8%)	0.46
**Heme+Neuro+Renal**	497 (6.3%)	140 (28.2%)	0.84	178 (17.8%)	0.57
**Heme+Neuro+Resp**	817 (10.3%)	191 (23.4%)	0.62	298 (29.7%)	0.14
**Heme+Renal+Resp**	481 (6.1%)	146 (30.4%)	0.19	174 (17.4%)	0.05
**Immuno+Liver+Neuro**	257 (3.3%)	78 (30.4%)	0.58	105 (10.5%)	<0.001*
**Immuno+Liver+Renal**	196 (2.5%)	64 (32.7%)	0.57	83 (8.3%)	0.06
**Immuno+Liver+Resp**	226 (2.9%)	76 (33.6%)	0.32	103 (10.3%)	0.001*
**Immuno+Neuro+Renal**	517 (6.5%)	103 (19.9%)	0.10	179 (17.9%)	0.80
**Immuno+Neuro+Resp**	943 (11.9%)	162 (17.2%)	0.25	299 (29.8%)	0.90
**Immuno+Renal+Resp**	484 (6.1%)	110 (22.7%)	0.03	176 (17.6%)	0.56
**Liver+Neuro+Renal**	274 (3.5%)	107 (39.1%)	0.18	101 (10.1%)	0.80
**Liver+Neuro+Resp**	362 (4.6%)	141 (39.0%)	0.44	133 (13.3%)	<0.001*

**Liver+Renal+Resp**	256 (3.2%)	105 (41.0%)	0.05	102 (10.2%)	0.64
**Neuro+Renal+Resp**	721 (9.1%)	170 (23.6%)	0.94	236 (23.6%)	0.17

*p<0.005 considered statistically significant.

Coag: coagulation
CV: cardiovascular
Endo: endocrinologic
Immuno: immunologic
Heme: hematologic
Neuro: neurologic
Resp: respiratory

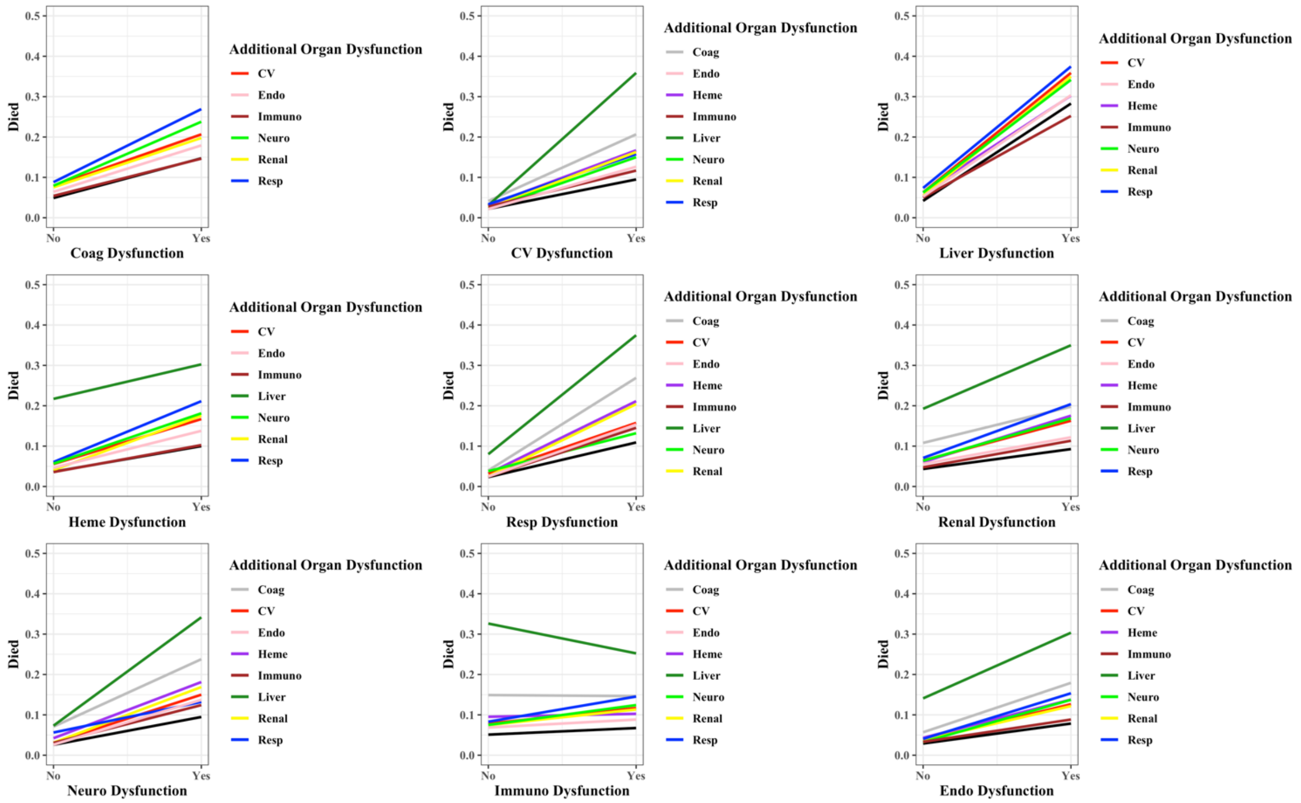

Supplementary Figure 1. Interaction plots for two organ dysfunctions and association with mortality. Black line represents single organ dysfunction, colored lines (per legend) represent interaction with second organ dysfunction. *Coag*: coagulation**;** *CV*: cardiovascular**;** *Endo*: endocrinologic**;** *Immuno*: immunologic**;** *Heme*: hematologic**;** *Neuro*: neurologic**;** *Resp*: respiratory

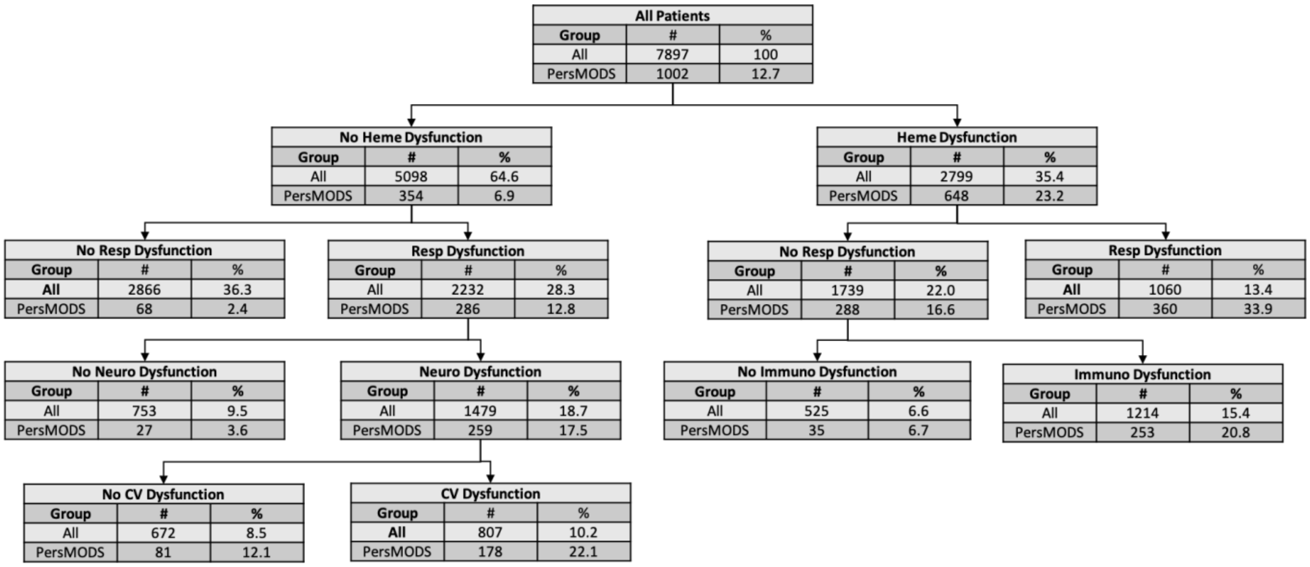

Supplementary Figure 2. Classification Tree for Persistent MODS using 10-fold cross validation. The top node of the decision tree, the root node, shows the total number of subjects in the cohort and the number and proportion of non-survivors. Each subsequent node shows the criterion for subsequent decisions, along with the number and proportion of patients with or without the organ dysfunction and the proportion who died. Terminal nodes show the risk for an individual with the preceding organ dysfunctions. AUROC = 0.74 (95% CI 0.70-0.78). *Coag*: coagulation**;** *CV*: cardiovascular**;** *Endo*: endocrinologic**;** *Immuno*: immunologic**;** *Heme*: hematologic**;** *Neuro*: neurologic**;** *Resp*: respiratory
